# Cellular and molecular mechanisms of Notch signal in pulmonary microvascular endothelial cells after acute lung injury

**DOI:** 10.1590/1414-431X2023e12888

**Published:** 2023-12-22

**Authors:** Zheng Yang, Jilin Ma, Zhihui Li, Jie Wang, Zhanli Shi

**Affiliations:** 1 Department of Intensive Care Unit, Hangzhou Red Cross Hospital, Hangzhou, Zhejiang, China; 2 Department of Rheumatism and Immunology, Hangzhou Red Cross Hospital, Hangzhou, Zhejiang, China

**Keywords:** Acute lung injury (ALI), Notch, Inflammatory response, Th17, Treg, Autophagosome

## Abstract

This study focused on the effect and mechanism of Notch signal on pulmonary microvascular endothelial cells (PMVECs) following acute lung injury. PMVECs were cultured *in vitro* and randomly divided into eight groups. Grouping was based on whether cells were co-cultured with T cells (splenic CD4^+^T cells were isolated using MACS microbeads) and the level of Notch expression: Normal group and Normal+T cells group, Model group and Model+T cells group, Notch low-expression group and Notch low-expression+T cells group, and Notch overexpression group and Notch overexpression+T cells group. Except for the Normal group and Normal+T cells group, all other groups were treated with 500 μL lipopolysaccharide (1 μg/mL). The expression of VE-cadherin and Zo-1 protein in the Model group (with or without T cells) was lower than that in the normal group (with or without T cells), their expression in the Notch low-expression group (with or without T cells) was significantly increased, and their expression in the Notch overexpression group (with or without T cells) was significantly decreased. Compared with the normal+T cells group, the number of Treg cells in the Notch low-expression+T cells group decreased significantly (P<0.01). The number of Th17 cells in the Notch overexpression+T cells group was higher than that in the Model+T cells group (P<0.01), while the number of Treg cells decreased (P<0.01). Our results demonstrated that activated Notch signal can down-regulate the expression of the tight junction proteins VE-Cadherin and Zo-1 in PMVECs and affect Th17/Treg immune imbalance. Autophagy was discovered to be involved in this process.

## Introduction

Acute lung injury (ALI) is caused by various non-cardiogenic pathogenic factors inside and outside the lung, such as severe infection, trauma, disseminated intravascular coagulation, shock, and other diseases. The clinical manifestations are acute progressive dyspnea and refractory hypoxemia, which are common critical diseases in the clinic.

Infection is considered to be the common cause of ALI in clinical patients. At present, it is considered that the imbalance between pro-inflammatory cytokines and anti-inflammatory cytokines in the process of infection is the key link in the occurrence and development of ALI, and the change of T lymphocytes is precisely the immunological core of the pro-inflammatory/anti-inflammatory imbalance (1). Helper T (Th)17 and Treg cells are newly discovered CD4^+^T lymphocytes that are different from Th1 and Th2 cells and play an important role in the occurrence and development of inflammation. It has been found that Th17 cells mediate the inflammatory response by secreting pro-inflammatory factors such as interleukin (IL)-17A. Treg cells are cells with immunosuppressive function, which play an inhibitory role through cell-cell contact or secretion of cytokines (such as IL-10 and tumor growth factor (TGF)-β). In normal state, TGF-β stimulation of initial CD4^+^T (naive CD4^+^T) expression of Foxp3 mediates immune tolerance, but in the inflammatory state, the body produces IL-6 and TGF-β by inducing the expression of retinoic acid related orphan nuclear receptors (RORγt), which promotes the differentiation of initial CD4^+^T cells into Th17 cells, thereby mediating the inflammatory response (2). Therefore, Th17 and Treg cells inhibit and transform each other, and play an important role in the pro-inflammatory/anti-inflammatory balance of the body.

The imbalance between CD4^+^CD25^+^Foxp3^+^ regulatory T (Treg) cells and Th17 cells has been found in a variety of inflammatory and autoimmune diseases (3,4), Th17/Treg imbalance also plays an important role in the occurrence and development of ALI. Th17/Treg cell ratio can be used as a risk indicator of ALI (5). However, the current research on Th17/Treg balance in ALI mostly focuses on alveolar epithelium, and the effect and mechanism of Th17/Treg immune imbalance on pulmonary microvascular endothelium are still unclear. From this, we speculated that the expression level of Notch signals during ALI is related to the degree of damage to PMVECs, and that Th17/Treg immune imbalance is involved in the process of PMVECs damage. Therefore, we used lipopolysaccharide (LPS) for cytological experiments to explore the effect of Th17/Treg immune imbalance on changes in pulmonary microvascular endothelial permeability, and demonstrated in-depth the effect of Notch signal on Th17/Treg immune balance, so as to further clarify the cellular immunological mechanism of pulmonary microvascular endothelial permeability changes during ALI.

## Material and Methods

### Culture of PMVECs

Mouse pulmonary microvascular endothelial cells (PMVECs) (product No. mic-cell-0001), special basic culture medium for primary endothelial cells (product No. med-0002), and special additives (product No. sup-0002) were purchased from Wuhan Primary Biomedical Technology Co., Ltd., China. All procedures were approved by the Animal Ethic Committee of Hangzhou HIBIO Co., Ltd., China (IACUC protocol number: HBFM3.68-2015).

### Chemicals

LPS was purchased from Beyotime Biotechnology (s1732, 0.5 mg, China) and fetal bovine serum was purchased from Bi Company (04-001-1acs, Israel). Both the Notch high expression lentivirus vector and the Notch small interfering RNA lentivirus vector were constructed by Hanheng Biotechnology Co., Ltd. (China). Zo-1 (ab190085) and VE-Cadherin (ab205336) were purchased from Abcam Co. (USA). Goat anti-rabbit IgG (H+L) cross-adsorbed secondary antibody, Alexa fluor 555 (a-21428), donkey anti-goat IgG (H+L) cross-adsorbed secondary antibody, and Alexa fluor 647 (a-21447) were purchased from Invitrogen (USA). Naive CD4^+^ T cell isolation kit (130-104-453) was purchased from Miltenyi Biotec (Germany). FITC anti-mice CD4 antibody (100406) and PE anti-mice CD25 antibody (101904) were both purchased from Biolegend (USA) and FOXP3 monoclonal antibody and PE-Cyanine7 (25-5773-82) were purchased from Invitrogen.

### Experimental protocol

Mouse PMVECs were cultured in the culture medium at 37°C and 5% CO_2_ in a saturated humidity incubator. When the density of PMVECs reached 90%, the culture medium was removed and cells were washed twice with PBS for later use. Then, complete culture medium was added to terminate digestion after trypsin digestion for 2 min. The cells were transferred to a 15-mL centrifuge tube and centrifuged at 120 *g* for 5 min at room temperature. Cells were placed onto a 24-well plate (5×10^4^ cells per well), incubated overnight, and grouped when cell density was 90%. At the same time, naive CD4^+^T cells from the spleen of C57BL/6 mice were separated by magnetic-activated cell sorting (MACS).

PMVECs were randomly divided into eight groups as follows: 1) Normal group (CK group): PMVECs were cultured normally for 24 h; 2) Normal+T cells group (CK+T cells group): PMVECs and C57BL/6 mouse spleen naive CD4^+^T cells were co-cultured for 24 h, PMVECs were grown on the lower surface of a transwell membrane, and CD4^+^T cells were placed on the upper surface of the transwell membrane at a PMVECs:naive ratio of 1:3 (same co-culture method for all groups below); 3) Model group (M group): PMVECs were treated with 500 μL LPS (1 μg/mL) for 24 h; 4) Model+T cells group (M+T cells group): PMVECs were treated with 500 μL LPS (1 μg/mL) for 24 h and then co-cultured with naive CD4^+^T cells for 24 h; 5) Notch low-expression group (Notch KD group): according to CBFl/RBP-Jĸ Sequence (Gene bank) instructions, small interfering RNA (siRNA) retroviral vector was constructed and transfected into PMVECs through cell culture; total RNA was extracted from cells, and the silencing efficiency was detected by RT qPCR. The others were the same as Model group; 6) Notch low-expression+T cells group (Notch KD+T cells group): according to CBFl/RBP-Jĸ Sequence (Gene bank) instructions, siRNA retroviral vector was constructed and transfected into PMVECs through cell culture; total RNA was extracted from cells, and the silencing efficiency was detected by RT qPCR. The others were the same as the Model+T cells group; 7) Notch overexpression group (Notch OE group): mouse intracellular domain of Notch1 (ICN1) gene was cloned, a retroviral vector carrying ICN1 was constructed, and was transfected into PMVECs through cell culture. The others were the same as the Model group; 8) Notch overexpression+T cells group (Notch OE+T cells group): mouse ICN1 gene was cloned and a retroviral vector carrying ICN1 was constructed and transfected into PMVECs through cell culture. The others were the same as Model+T cells group.

**Figure 1:**
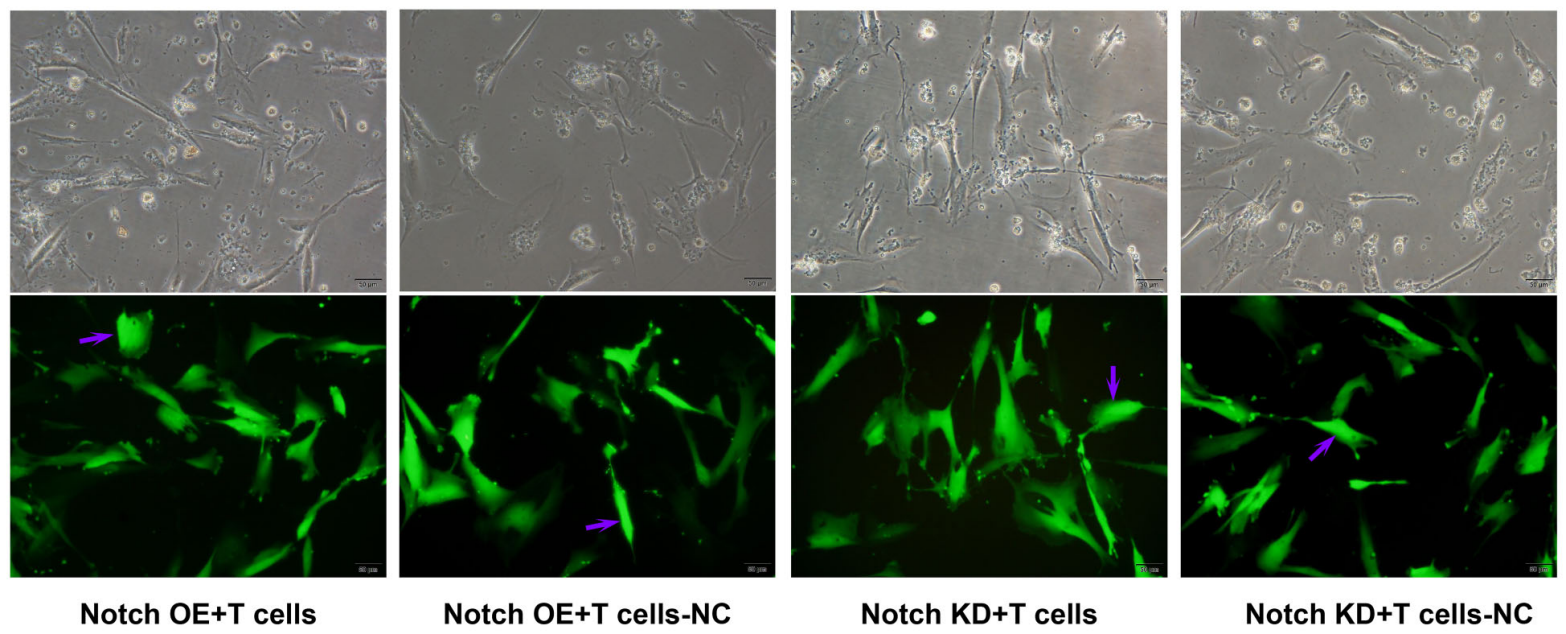
Inverted microscope images (top) and fluorescence expression (bottom) of stable lentivirus strain in mouse pulmonary microvascular endothelial cells (light microscope ×20 magnification, scale bar 50 μm). NC: normal control; OE: overexpression; KD: low-expression. Purple arrow: green fluorescent protein.

Construction method of siRNA: after cells are seeded, when cell coverage reaches 50%, the culture medium in the plate is discarded and the culture medium replaced. When the multiplicity of infection (MOI) value is 10, 20, 30, and 40, different amounts of virus volume (added virus volume = cell volume MOI × cell number / virus titer × 1000) are added onto the corresponding well plate. The plate is placed into the incubator, the solution is changed after virus infection for 24 h, and cell fluorescence is observed. When the MOI value = 30, the infection efficiency of each group reached more than 95%. The inoculation was expanded according to this proportion, and the culture was expanded normally. Pteromycin at 2 μg/mL was used to screen and purify the cells and construct stable transgenic strains. Green fluorescent protein (GFP) indicated that siRNA modeling was successful. The results are shown in Figure 1.

### Cell morphology and structure

PMVECs were cultured under different conditions before being trypsinized and fixed in 2.5% glutaraldehyde. Subsequently, cells were post‐fixed in 1% osmium tetroxide with 0.1% potassium ferricyanide, dehydrated through a graded series of ethanol (50-100%), and embedded in epoxy resin. Samples were cut into ultrathin sections (60-80 nm). Ultrathin sections were stained with 2% uranyl acetate saturated alcohol solution and lead citrate, and the images were acquired using a TECNAI-10 transmission electron microscope (Royal Dutch Philips Electronics Ltd., the Netherlands). Images were used to observe autophagy and mitochondrial morphology.

### VE-Cadherin and zonula occludens 1 (Zo-1) in vascular endothelial cells

Immunofluorescence was used to detect the distribution and expression of VE-Cadherin and zonula occludens. The specific methods were as follows: Cells were washed with PBS 3×3 min and fixed with 4% paraformaldehyde at room temperature for 20 min, followed by PBS washing 3×3 min. Cells were incubated with 0.1% Triton X-100 at room temperature for 20 min, and then washed with PBS 3×3 min. Cells were incubated with 5% BSA at room temperature for 30 min, and then washed with PBS 1×3 min. Primary antibodies for VE-Cadherin and Zo-1 were added overnight at 4°C, and washed with PBS 3×3 min. Alexa flow secondary antibody was added and incubated at room temperature in the dark for 1-2 h, and then washed with PBS 3×3 min. Then, 10 ng/mL DAPI (1:100-1:500) was added and incubated at 4°C in dark for 15-30 min, and then washed with PBS 3×3 min.

### Number of Th17 and Treg cells

Harvested CD4 T cells were counted for Th17 and Treg percentages using flow cytometry (BD Accuri C6 Software, USA). Before detection of Th17 cells, cells were stimulated for 4 h with 2 μL/mL leukocyte activation cocktail (BD Pharmingen™, USA) at 37°C and 5% CO_2_. Cells were stained with anti-mouse CD4 antibody (Biolegend). Afterwards, cells were fixed, permeabilized, and labelled with anti-mouse IL-17A (Biolegend). Th17 were cells which expressed CD4 IL-17A. For detection of Treg, cells were labelled with anti-mouse CD4 antibody (Biolegend) and anti-mouse CD25 antibody (Biolegend). Anti-mouse Foxp3 antibody (eBioscience™, Invitrogen) was added after cells were fixed and permeabilized. Treg were cells that expressed CD4 CD25 Foxp3.

### Statistical analysis

Statistical analyses were performed using the Statistical Package for Social Science (SPSS) for Windows (Version 22.0 software, SPSS Inc., USA) and GraphPad Prism software, version 7 (GraphPad, USA). Differences between two groups were analyzed using Student's *t*-test or Mann-Whitney U-test, as applicable. Differences between multiple groups were analyzed by one-way ANOVA and chi-squared test. P-values less than 0.05 were considered significant. Data are reported as means±SD.

## Results

### Morphology and structure of pulmonary capillary cells

#### Inverted microscope

**Figure 2:**
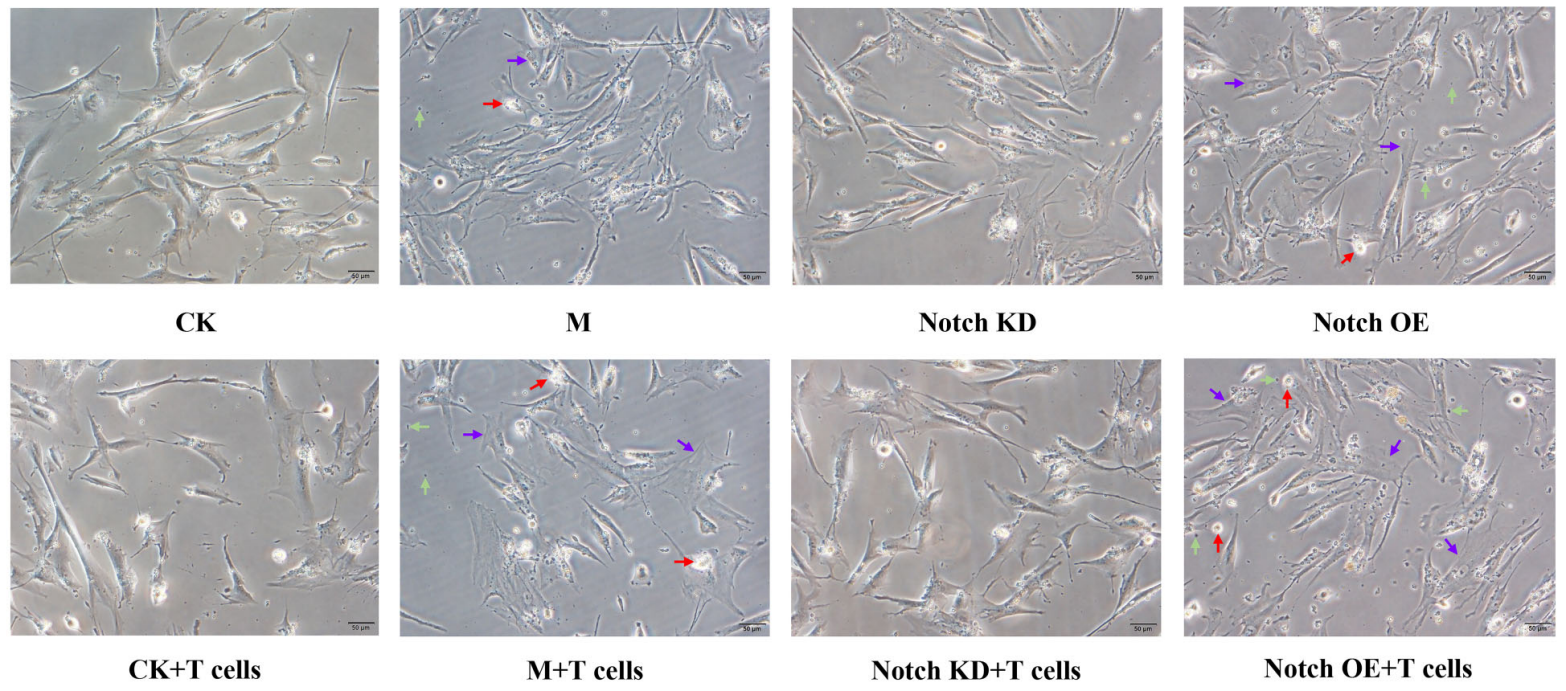
Morphology and structure of capillary cells under inverted microscope. Purple arrow: thinner cells; red arrow: decreased refractive index of a cell and increased transparency; green arrow: cell debris. Scale bar 50 μm. CK: Normal; M: Model control; Notch KD: Notch low-expression; Notch OE: Notch overexpression.

Compared with the CK+T cells group, the cell stereopsis of PMVECs in the M+T cells group was worse, nuclear cavitation or nuclear fragmentation was visible, and fragments (small black spots) increased. The refractive index of cells was worse, and the cells became thinner. The adherence of cells was poor and easy to detach. Compared with M+T cells group, the cell problems in Notch KD+T cells group were reduced and aggravated in the Notch OE+T cells group. Changes in the groups with T cells were more serious than that of the corresponding groups. The results are shown in Figure 2.

#### Transmission electron microscope

**Figure 3:**
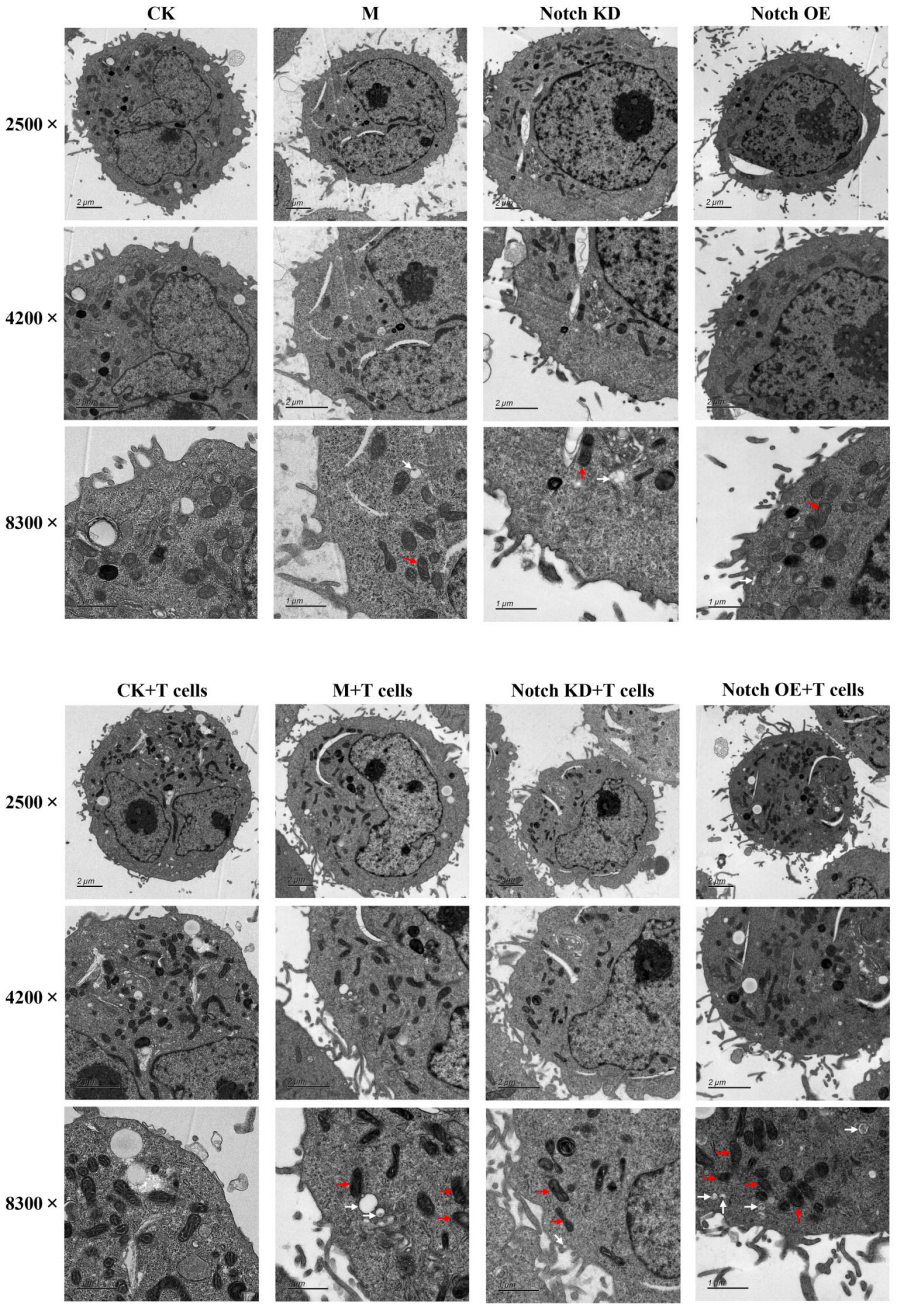
The morphology and structure of pulmonary microvascular endothelial cells were detected by transmission electron microscope (using magnifications of ×2500, ×4200, and ×8300, scale bars 2 μm, 2 μm, and 1 μm, respectively). Red arrow: mitochondria; white arrow: autophagy bodies. CK: Normal; M: Model control; Notch KD: Notch low-expression; Notch OE: Notch overexpression.

In the CK+T cells group, the three-layer structure of the cell membrane was obvious, the mitochondrial structure was complete, and the cristae were clearly visible. Compared with the CK+T cells group, the three-layer structure of the M+T cells group was incomplete, with swelling of mitochondria, disappearance of cristae, and an increase of autophagic bodies. Compared with the M+T cells group, the Notch KD+T cells group showed incomplete membrane structure, less swelling of mitochondria, and less autophagic bodies. In the Notch OE+T cells group, the structure of the cell membrane was seriously damaged, the mitochondria was significantly swelled, the cristae disappeared, and there were more autophagic bodies. The damage of the groups with T cells was more severe than that of the corresponding groups. The results are shown in Figure 3.

### VE-Cadherin and Zo-1 distribution and expression

**Figure 4:**
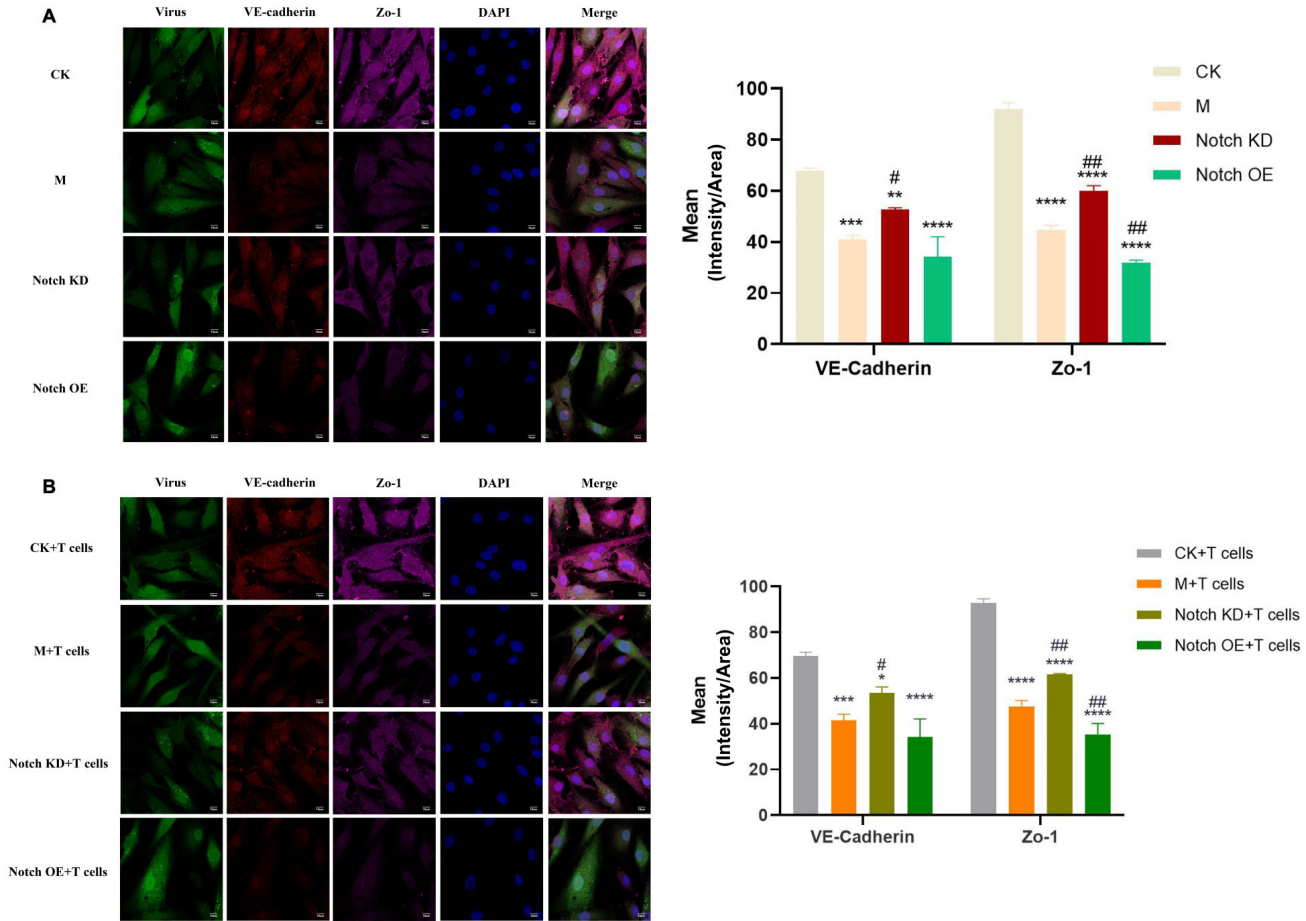
Immunofluorescence assay was used to detect the distribution and expression of VE-Cadherin and Zo-1. The data are reported as means±SD. **A**, ^#^P<0.05, ^##^P<0.01 *vs* M; ***P<0.001, ****P<0.0001 vs Notch KD. **B**, *P<0.05, ***P<0.001, ****P<0.0001 *vs* CK+T cells; ^#^P<0.05, ^##^P<0.01 *vs* M+T cells (ANOVA). DAPI: development of staining solution of living cells and fixed cells; Merge: pulmonary microvascular endothelium is developed after DAPI staining. Scale bars: 10 μm. CK: Normal; M: Model control; Notch KD: Notch low-expression; Notch OE: Notch overexpression.

Compared with the CK+T cells group, the expression of VE-Cadherin and Zo-1 protein in M+T cells group was decreased. Compared with the M+T cells group, the expression of the two proteins in the Notch KD+T cells group increased, while the expression of the two proteins in Notch OE+T cells group decreased significantly. There was no significant statistical difference in the expression of these two proteins between the groups without T cells and the corresponding groups (with T cells). The results are shown in Figure 4.

### Number of Th17 and Treg cells

Compared with the normal control group, the number of Th17 cells in the Model group increased significantly (P<0.01). Th17 cells in the Notch low-expression group were significantly lower than those in the Model group (P<0.01), while Th17 cells in the Notch overexpression group were significantly higher than those in the Model group (P<0.01).

**Figure 5:**
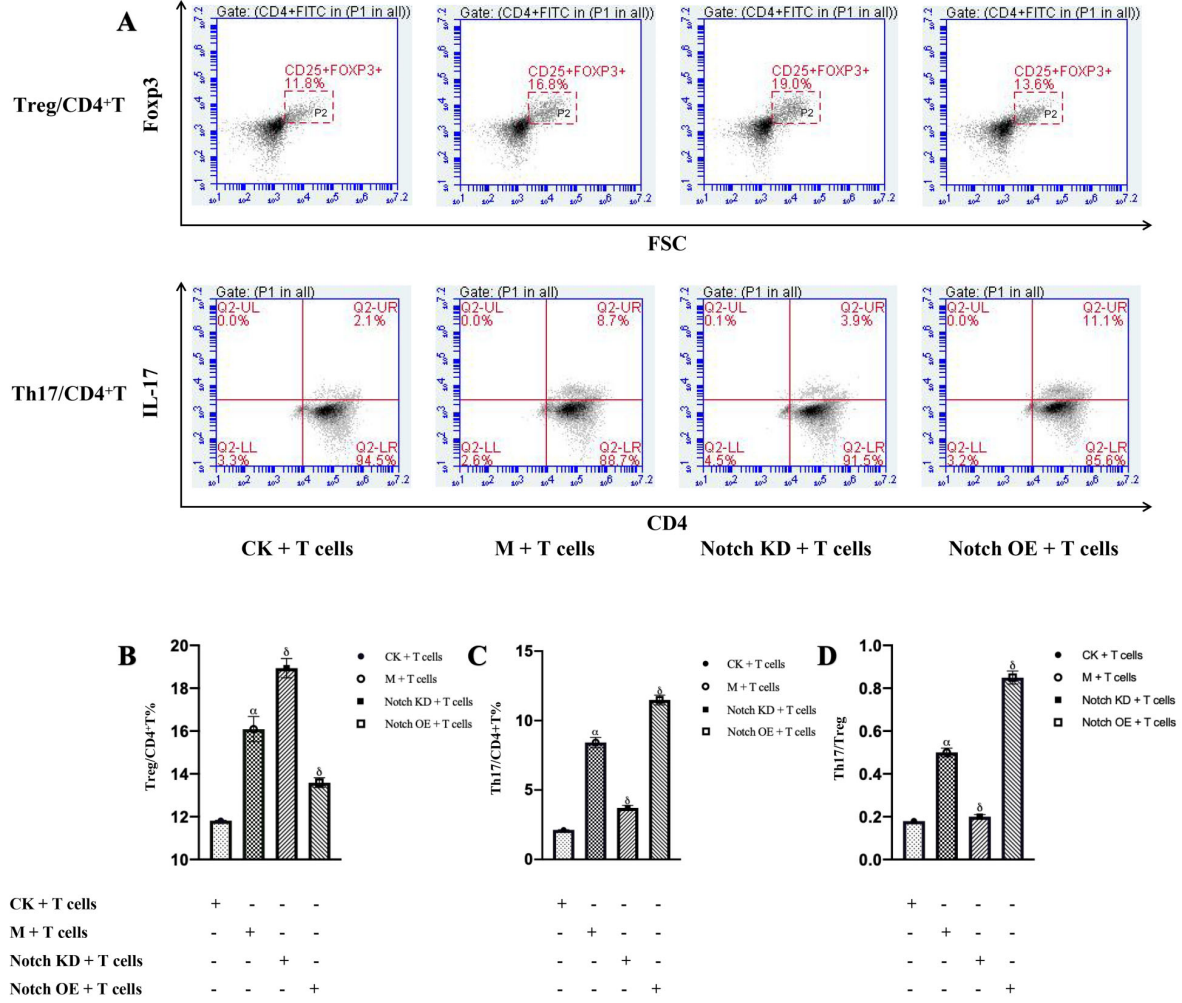
**A**, Flow cytometry was used to determine the percentages of Treg and Th17 cells differentiated from naive CD4+T cells: the horizontal axis is CD4 positive, the vertical axis is IL-17 positive, and the Q2-UR part indicates CD4, IL-17 positive, that is, the number of Th17 cells. The number of Treg cells detected by flow cytometry: the horizontal axis is CD25 positive, the vertical axis is Foxp3 positive, and the P2 part indicates CD4, CD25, and Foxp3 positive, that is, the number of Treg cells. **B**, Percentages of Treg cells. **C**, Percentages of Th17 cells. **D**, Th17/Treg cell ratios. The data are reported as means±SD. ^α^P<0.01 *vs* CK+T cells condition, ^δ^P<0.01 *vs* M+T cells condition (ANOVA). CK: Normal; M: Model control; Notch KD: Notch low-expression; Notch OE: Notch overexpression.

Compared with the normal control group, the number of Treg cells in the Model group, Notch low-expression group, and Notch overexpression group increased significantly (P<0.01). Compared with the Model group, the number of Treg cells in the Notch low-expression group increased significantly (P<0.01), while the number of Treg cells in the Notch overexpression group decreased significantly (P<0.01). The results are shown in Figure 5.

## Discussion

In our study, overexpression of Notch exacerbated membrane structural damage, caused significant mitochondrial swelling, disappearance of cristae, and increased autophagy, and reduced the expression of VE-Cadherin and Zo-1 proteins. The number of Th17 cells in the Notch overexpression+T cells group was greater than that in the Model+T cells group, and the number of Treg cells decreased, while the Notch low-expression group obtained the opposite result. Activated Notch signal can down-regulate the expression of the tight junction proteins VE-Cadherin and Zo-1 in PMVECs, participate in the pathological mechanism of acute lung injury, and affect Th17/Treg immune imbalance. In this process, increased autophagy was involved.

Notch signaling molecules are a very important family of transmembrane signaling receptor proteins in the development of multicellular organisms, which are widely involved in immune and inflammatory responses. Our previous study on a mouse model of severe acute pancreatitis found that, in the early stage of ALI (within 24 h), the expression of Notch signal in lung tissue is inhibited, resulting in the reduction of apoptosis in lung tissue and aggravating the process of lung injury, but the expression of Notch signal increases at 24 h (6). A study using yeast glycan to induce ALI in a mouse model found that Notch signal activity in lung tissue increases 6 h after modeling and peaks at 24 h, confirming that the inhibition of Notch signal can reduce lung injury (7), which is consistent with the results of this study. Notch signaling plays an important role in inflammatory response by controlling the activation and differentiation of initial naive CD4^+^T cells into different surrounding subpopulations (8). Notch signal has a “bridging” effect between effectors of T cell metabolism through DLL4 and Jagged1 and other ligands, and it regulates vesicle transport between cell membranes, thus regulating Th17 differentiation and controlling the migration of IL17 and metabolic regulators (9-11). *In vivo* studies have preliminarily found that inhibiting Th17 cell response by blocking Notch signal can reduce LPS-induced ALI in mice (12). This *in vitro* experiment further confirmed that the number of Th17 and Treg cells in PMVECs in the Model group was significantly higher than that in the normal group. The proportion of Th17 cells decreased significantly and the proportion of Treg cells increased significantly when Notch1 was low-expressed, while the proportion of Th17 cells increased significantly and the proportion of Treg cells decreased significantly when Notch1 was overexpressed. Therefore, it was confirmed that Notch signal has regulatory significance in the Th17/Treg immune balance during ALI. The enhanced expression of Notch 1 can promote the differentiation of Th17 cells and inhibit the differentiation of Treg cells. This regulation aggravates the inflammatory response of PMVECs and participates in the pathogenesis of ALI.

Microvascular endothelial cells, alveolar epithelial cells, and tight junctions form the alveolar capillary barrier. Inflammatory reaction leads to the injury of pulmonary vascular endothelial cells, which is the pathological basis of ALI. Pulmonary edema caused by the change of pulmonary vascular endothelial permeability is the pathological feature of ALI. Tight junctions are an important component of the capillary alveolar barrier and are essential to maintain structural integrity. VE-Cadherin is a key molecule of vascular endothelial cell adhesion and connection. Its main function is to promote the adhesion between homologous cells and maintain endothelial stability. In addition, it is also involved in endothelial cell biological reactions such as contact inhibition of cell growth, prevention of apoptosis, and control of endothelial permeability (13). Zo-1 is one of the important components of tight junction, participates in the formation of cytoskeleton, and is a marker of tight junction stability and integrity. Previous studies have suggested that plasma Zo-1 protein level is a valuable prognostic biomarker of sepsis severity, its expression is related to infection, and is a predictor of 30-day mortality in patients with sepsis (14). Many studies have confirmed that the down-regulation of the expression of VE-Cadherin and Zo-1 proteins is involved in the changes of pulmonary microvascular permeability during ALI (15,16). Unfortunately, the specific regulatory mechanism is still unclear. In a study on cardiovascular development, it was pointed out that Notch activation in vascular endothelial cells can lead to consistent morphological, phenotypic, and functional changes in mesenchymal transformation, including the down-regulation of vascular VE-Cadherin and other endothelial markers (α-smooth muscle actin, fibronectin, and platelets), the up-regulation of mesenchymal markers, and the migration of platelet-derived growth factor BB. It was concluded that the Jagged1-Notch interaction induces endothelial-to-mesenchymal transformation (17). Another experiment on the epithelial mesenchymal transformation of pulmonary fibrosis type II alveolar epithelial cells found that, compared with the control group, the alveoli in the experimental group shrank, collapsed, and fused, the alveolar septum was significantly widened, and a large number of inflammatory cells infiltrated the pulmonary stroma (18). *In vivo* and *in vitro* studies of the experimental group showed that the expression of Zo-1 and VE-Cadherin decreased, accompanied by the increased expression of Notch-1, NICD, HES-1, and twist-1, while the expression of VE-Cadherin and Zo-1 increased significantly after treatment with Notch-1 siRNA (18).

Our study found that the morphological and structural integrity of PMVECs in the Model group was damaged and further worsened with the enhancement of Notch signal, with nuclear cavitation or fragmentation and mitochondrial damage, but the inhibition of Notch signal reduced the pathological phenomenon, suggesting that Notch signal pathway plays an important role in the structural changes of PMVECs in ALI. This study observed that the fluorescence intensity of the Notch overexpression group was significantly lower than that of the Model control group, while the fluorescence intensity of VE-Cadherin and Zo-1 protein in the low-expression group was higher than that of the Model group, suggesting that Notch signal expression significantly inhibited the expression of VE-Cadherin and Zo-1 protein during ALI, thereby increasing the permeability of pulmonary capillary barrier. However, a study (18) found that atypical Notch signals mediated by blood flow can promote barrier integrity. Blood laminar shear stress promotes the expression of Notch ligand DLL4 and the cleavage of Notch receptor. Under the interaction of ligand receptor, the extracellular domain (ECD) of Notch 1 is cleaved by G secretase and the intracellular domain (ICD) of Notch 1 is released. The remaining transmembrane domains form protein complexes with the adhesive band protein VE-Cadherin, tyrosine phosphatase LAR, and guanylate exchange factor trio. The latter activates Rac1 to stabilize adhesion connections, thereby promoting the formation of vascular endothelial barrier. This non-classical Notch signaling mechanism is different from the traditional role of Notch1 in transcriptional regulation, highlighting its new function as a mechanical sensor. Flow-dependent Notch1 activation controls permeability in a fast, transcription-independent manner, whereas additional anti-inflammatory effects are mediated by canonical transcription-dependent signaling (19). This shows that in inflammation-related ALI, blood laminar shear stress also has a certain effect on Notch signal, and the influence of this factor needs to be further considered with *in vivo* experimental research.

Macrophage autophagy (hereinafter referred to as autophagy) is an intracellular lysosome-dependent degradation system. Through this degradation system, misfolded proteins and dysfunctional organelles are transported to autophagosomes, which then fuse with lysosomes, and the cytoplasmic contents are circulated and digested. Recently, it has been reported that LPS can induce autophagy in PMVECs. Relevant studies found that LPS treatment reduced the expression of Zo-1, which was further aggravated by the inhibition of autophagy in advance, suggesting that autophagy has a certain protective effect on maintaining the integrity of microvascular endothelial barrier in LPS-induced lung injury (20). However, the occurrence and regulatory mechanism of autophagy during ALI are not clear at present. Our study explored this mechanism and found that autophagy corpuscles of PMVECs in the Model group increased, suggesting that the destruction of proteins and organelles of endothelial cells increased. At the same time, the autophagy corpuscles increased significantly when Notch was overexpressed and decreased when Notch was low-expressed, suggesting that the expression of Notch signal also has a certain impact on the autophagy of PMVECs. This may be related to the increased expression of Notch, which aggravates the injury of PMVECs, and thus leads to the increase of autophagy corpuscles.

Recent studies have confirmed that the content of Th17 cell-related factors and the ratio of Th17/Treg cells are increased in the alveolar lavage fluid of both clinical patients and experimental animals with ALI (21-24), while the highly restrictive defect of Th17 cells, the deletion of IL-17 receptor, or the blocking of IL-17A antibody can significantly reduce ALI (25,26). Several studies have also found that Treg cells can secrete TGF in the lungs, inducing neutrophil apoptosis and other mechanisms to reduce ALI (27). In the later stage, Tregs can also promote the proliferation of alveolar type II epithelial cells and play an important role in the repair of lung tissue (28). On the contrary, the decrease of Treg cell aggregation in lung tissue leads to the decrease of IL-10 synthesis, which further aggravates ALI (29). The above studies show that Th17/Treg immune balance is involved in the pathogenesis of ALI.
